# Incidence and risk factors of postoperative nausea and vomiting in Africa among patient under gone surgery: a systematic review and meta-analysis

**DOI:** 10.1097/MS9.0000000000002845

**Published:** 2025-01-09

**Authors:** Mebratu Tila Bacho, Getahun Dendir Wolda, Wondu Reta Demssie, Nefsu Awoke, Ashagrie Sintayehu Temsgen, Elias Habtu Sulieman, Tsegaye Demeke Geberemedin, Ashebir Debalkie Gemechu, Naol Gorde Wosene, Andualem Assefa Endebirku, Wogayehu Abera Negash, Afwork Alemu, Alemu Urmle Kussie, Wondafrsh Kusie, Mohammed Suleiman Obsa

**Affiliations:** a College of Health Science and Medicine, School of Anesthesia, Wolaita Soddo University, Soddo, Ethiopia; b College of Medicine and Health Science, School of Nursing, Wolaita Soddo University, Soddo, Ethiopia; c College of Medicine and Health Science, Jimma University, Jimma, Ethiopia; d College of Medicine and Health Science, Department of Anesthesia, Arsi University, Asela, Ethiopia

**Keywords:** incidence, nausea, postoperative, surgery, vomiting

## Abstract

**Background:**

Patients who experience postoperative nausea and vomiting are not happy with their surgical outcomes. Preventing this problem expedites the patients’ return to normal activities following surgery and significantly increases their satisfaction. There are no condensed results that show the prevalence and contributing variables of postoperative nausea and vomiting in Africa. Thus, the purpose of this meta-analysis and comprehensive review was to ascertain the prevalence and contributing variables of postoperative nausea and vomiting in Africa.

**Methods:**

Studies were retrieved from the PubMed, EMBASE, Cochrane Database, CINAHL, Scopus, Mednar, and Google Scholar databases using combinations of searching terms and Boolean operators. *I*-squared (*I*^2^) statistics is used for evaluating study heterogeneity. Every publication is assessed for methodological quality using the Joanna Briggs Institute (JBI) Critical Appraisal criteria. Using a funnel plot, publication bias is visually assessed. Subgroup analyses investigate the source of heterogeneity. To determine whether publication bias exists, the Eggers weighted regression test is employed. STATA software version 14 was used to perform statistical analyses.

**Results:**

In Africa, the combined incidences of nausea 24.96 % (95% CI: 17.903-32.018), vomiting 23.655 % (95% CI: 17.542-29.769) and nausea with vomiting 15.27 % (95% CI: 9.118-21.424) . History of motion sickness (odds ratio [OR]: 3.19 (95% CI 1.08–9.42), *P* < 0.036) and history of postoperative nausea and vomiting (OR: 4.33 (95% CI 2.654–7.07), *P* < 0.001) were factors linked to postoperative nausea and vomiting. Compared to their counterparts, patients who underwent more risky surgical procedures had a 1.4-fold increased chance of developing postoperative nausea and vomiting. Patients who skipped the use of prophylactic medication for nausea and vomiting had a 59% higher risk of experiencing postoperative nausea and vomiting than those who did [OR: 1.39 (95% CI (1.074–1.769), *P* < 0.012) and OR: 0.194 (95% CI (0.04–0.935), *P* < 0.001)], respectively.

**Conclusion:**

Postoperative nausea and vomiting were more common in surgically treated African individuals. Clinical interventions are needed to prevent, diagnose, and treat postoperative nausea and vomiting (PONV), with a focus on patients who have experienced motion sickness, high-risk surgery, or PONV in the past. It is advisable to use whole intravenous anesthesia based on Propofol for surgery, if feasible.

## Background

Vomiting is defined as the violent release of stomach or intestinal contents through the mouth, whereas nausea is defined as an uncomfortable feeling that is connected to the desire to vomit^[1]^. After surgery and anesthesia, nausea and vomiting that worsens within 24–48 hours is one of the most uncomfortable side effects that patients encounter^[2]^.

The vomiting center in the medulla oblongata is the main source of control over nausea and vomiting. It consists of the tractus solitarius nucleus and the reticular formation (NTS). Four main locations can provide signals to the vomiting center: the vestibular region, the cerebral cortex and thalamus, the gastrointestinal tract, and the chemoreceptor trigger zone (CRTZ)^[3]^. Indirect signals might also be sent to the vomiting center by irritations. The effects of anesthesia and surgery can have multiple contributing factors that lead to postoperative nausea and vomiting. Patient-related characteristics include young age, female sex, history of severe motion sickness, history of nausea and vomiting following surgery, and non-smoking history^[4]^.

Postoperative nausea and vomiting have been linked to a number of anesthesia-related risk factors, such as extended anesthesia duration, laparoscopic surgery, and the use of volatile anesthetics and opioids (both intra- and postoperative)^[5]^.

The frequency of postoperative nausea and vomiting has been documented in a number of European research; the prevalence ranges from 16.8% to 31.4%, with the first 24 hours having the highest frequency^[6]^.

Evidence from Ethiopia indicated that a study was carried out in Addis Ababa^[4]^ found that 54.3% of patients experienced postoperative nausea and vomiting. Both during the intraoperative and postoperative phases of the procedure, nausea and vomiting can happen. 40.2% of patients who underwent spinal anesthesia had this condition, according to a research done in Gonder, Ethiopia^[6]^. Adult patients’ risk of postoperative nausea and vomiting has been estimated using simplified risk score models, such as the Apfel score. It is assumed that the postoperative nausea and vomiting (PONV) risk is 10% in the absence of any risk factors. While postoperative opioid use and the presence of one of the four risk variables above is linked to a 20% risk of postoperative nausea and vomiting, the risk rises by 20% with each additional risk factor and reaches 80% if all four are present^[7]^.

The Apfel grading method does not take ethnicity into account, which may have an impact on the frequency of postoperative nausea and vomiting. The incidence of postoperative nausea and vomiting varied depending on the patient’s country of origin, according to a meta-analysis^[8]^.

Preventing postoperative nausea and vomiting is crucial because it can have negative psychological and physical effects in addition to leading to serious complications like upper airway and postoperative pulmonary complications from aspirating vomitus, pneumothorax, incisional hernia, delayed hospital discharge, and re-admission following outpatient surgery^[9,10]^. Additionally, it has a significant impact on lowering medical expenses, enhancing patient satisfaction, and expediting the return of patients to their regular activities^[11]^.

In order to improve postoperative nausea and vomiting care, a summary of the problem’s magnitude should be available; however, the results of research carried out in various regions of the nation now differ greatly from one another. Studies from Africa are not included in the worldwide systematic review and meta-analysis. Consequently, the purpose of this study was to show the pooled incidence of postoperative nausea and vomiting in Africa together with its contributing factors.

## Methods

### Study protocol and reporting

This systematic review and meta-analysis was carried out using the Preferred Reporting Items for Systematic Reviews and Meta-Analyses (PRISMA) standards for the literature search method, study selection, data extraction, and outcome reporting^[12]^. The JBI 2017 review guideline^[13]^, which established the Condition, Context, and Population (COCOPO) principle as the basis for eligibility, was modified.

The reference management programme EndNote (version X8) was utilized to download, arrange, examine, and cite relevant publications. This review’s protocol was entered into the Prospero database under the CRD 42022344024 reference number.

## Eligibility criteria

- Population: Surgical patients who reported experiencing nausea and vomiting following the procedure.
- Context: All research on the incidence, prevalence, and risk factors of postoperative nausea and vomiting in the African general population was taken into account.
- Condition: Studies measuring the outcome of interest based on incidence and/or risk factors of postoperative nausea and vomiting were taken into consideration for this review.
- Language: English-language studies are written.
- Study period: currently access to procedures/approaches for the prevention of postoperative nausea & vomiting improved, because of the current focus on enhanced recovery protocols for every surgery through multidisciplinary approaches and perioperative nausea & vomiting risk minimizing approaches due to advancements in medical equipment and medical personnel training. As a result, primary studies conducted more recently or published between 2018 and 2022 are included.
- Publication status: Both published and unpublished studies were included.

Exclusion criterion studies with inadequate methodology, variables, and measures.

The primary outcome of this study was postoperative nausea and vomiting.

## The case definition of postoperative nausea and vomiting

- Postoperative nausea is defined as nausea experienced within 24 hours after surgery but without accompanying vomiting.
- Postoperative vomiting: a 24-hour period following surgery during which vomiting alone occurs without accompanying nausea.
- Postoperative nausea and vomiting: a nausea symptom that appears 24 hours after surgery together with vomiting^[14]^.

### Search strategy

A preliminary narrow search of PubMed, THE EMBASE database, the Cochrane Database, The CINAHL website, the Scopus database, and AJOL for published studies. We also searched for gray or unpublished paper on Mednar, Google Scholar, different institution repositories and Google was conducted for unpublished studies. After that, a second search term was conducted using all of the discovered keywords and index terms. Third term, a search was conducted for more research studies in the reference list of all discovered papers and journals. We used search terms to look through the database.

### Data quality control measures

Every article’s methodological quality was assessed using the 2017 JBI Critical Appraisal checklist. Only studies having a minimum score of 7 or above for cross-sectional studies and a score of 9 or higher for cohort studies were included after each study was evaluated against these criteria^[15]^.

Two writers (M.T. and A.S.) independently assessed each study’s quality. After consulting with the third independent reviewer (G.D.), the disagreement was settled.

### Data extraction

Two independent writers (M.S. and W.R.) extracted the data using a standard and piloted form created in Microsoft Excel. The name of the author, the year the work was published, the nation, the research participant, the frequency of the illness, and risk variables are all included in the retrieved data. Conflicts were resolved through discussion and agreement, or by a third reviewer (E.H.).

### Statistical analysis

All statistical data analyses were performed using Stata version 14.0 (Statacorp. LP, College Station, USA). To compile data on postoperative nausea and vomiting in Africa, a random effect meta-analysis technique developed by DerSimonian and Laird was utilized. To ascertain whether statistical heterogeneity exists, Higgins *I*-squared (*I*^2^) statistics and the Cochran’s *Q* test were employed. The matching *I*-square values of 25%, 50%, and 75% were used to classify heterogeneity as low, moderate, or high^[16]^.

Meta-regression and subgroup analysis were employed to look at potential sources of heterogeneity. Cone plots were utilized to visually represent publication bias and test for it using the Egger’s and Begg tests^[17]^.

However, as the *P* value is higher than 0.05, the existence of unpublished studies (which support the hypothesis) is not supported statistically.

Sensitivity analysis was used to see how one study affected the estimate as a whole.

## Result

### Search results

**Figure 1.**
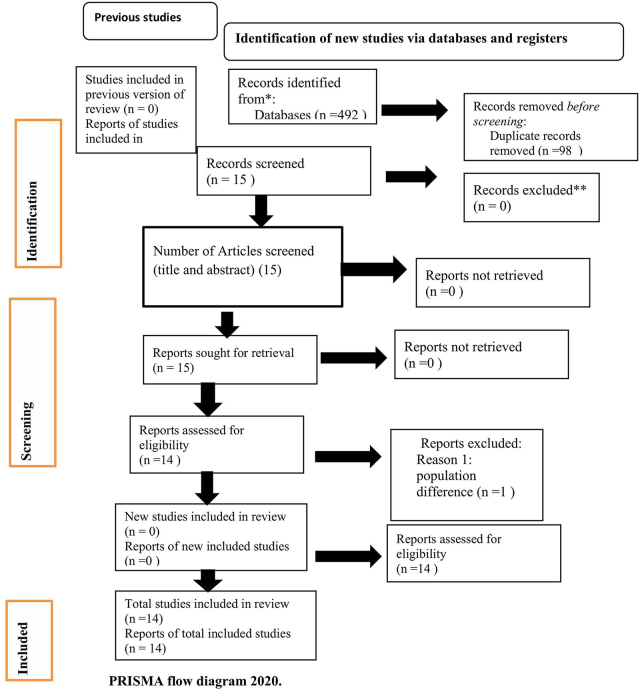
PRISMA flow diagram 2020.

In the beginning, 474 studies in total were obtained from the electronic databases. After establishing criteria for ten years, 394 articles were obtained. Nine papers were then chosen, and 18 articles were found by hand searching. 12 duplicates were identified and eliminated as a result. The titles and abstracts of the final six articles were screened. One study that was deemed unnecessary was eliminated. Ultimately, a total of 14 studies were included in the analysis after meeting the inclusion criteria (Fig. 1).

### Study characteristics

This systematic review and meta-analysis comprised 14 publications from African nations. 1036 patients experienced postoperative nausea and vomiting out of 4367 study participants. The studies’ sample sizes vary from 52^[16]^ to 800^[18,19]^. Of the fourteen articles, eight were from East Africa, six from Ethiopia, three from West Africa, two more from South Africa, and the remaining eight from Central Africa (Table 1).

**Table 1:** Characteristics of studies included in meta-analysis.

Authors name	Study area	Publication year	Design	Study duration	Sample	Frequency
N. Ahmed Abired *et al*	North Africa/Libya	2019	Cohort	6 months	170	44
Mengesha Dessie Allenea	East Africa/Ethiopia, Debrebran	2020	Cross-sectional	4 months	398	16
Chali Tolosa *et al*	East Africa/Ethiopia, Addis Ababa	2019	Cross-sectional	2 months	198	31
Ashagrie sintayehu *et al*	East Africa/Ethiopia, Wolita	2019	Cross-sectional	6 months	373	17
Petros K Yosief	East Africa/Erteria, Asmara	2022	Cohort	2 months	125	39
Queeneth N. Kalu, AtimI *et al*	West Africa/Nigeria	2015	Cohort	2 months	166	22
Reitze N, Rodseth *et al*	South Africa	2010	Cohort	4 months	800	135
Ashagrie sintayehu, Getahun Dendir	East Africa/Ethiopia	2019	Cross-sectional	6 months	373	108
Okafor UV *et al*	West Africa/Nigeria	2013	Cross-sectional	5 year retrospective	52	7
Okafor Ugochukwu *et al*	West Africa/Nigeria	2010	Cross-sectional	2 years retrospective	300	12
Seid Ahmed *et al*	East Africa/Ethiopia	2020	Cross-sectional	2 months	355	61
Dinkisisa Chamada *et al*	East Africa/Ethiopia	2019	Cross-sectional	2 months	368	101
Phillipo L .Chalya *et al*	East Africa/Tanzania	2015	Cohort	2 months	348	144
Endale G/Egziabher G/medhn, Jessica Hoyle *et al*	East Africa/Ethiopia	2012	Cross-sectional	2 months	509	97
Asfaw Nurhussen Fethiya Al’ferid *et al*	East Africa/Ethiopia	2020	Cross-sectional	2 months	154	68
Samson Mndolo *et al*	South East Africa	2014	Cohort		138	41

### Incidence of postoperative nausea and vomiting among patients who underwent surgery

To find the pooled effect size, a DerSimonian and Laird random effects model was fitted.

**Figure 2.**
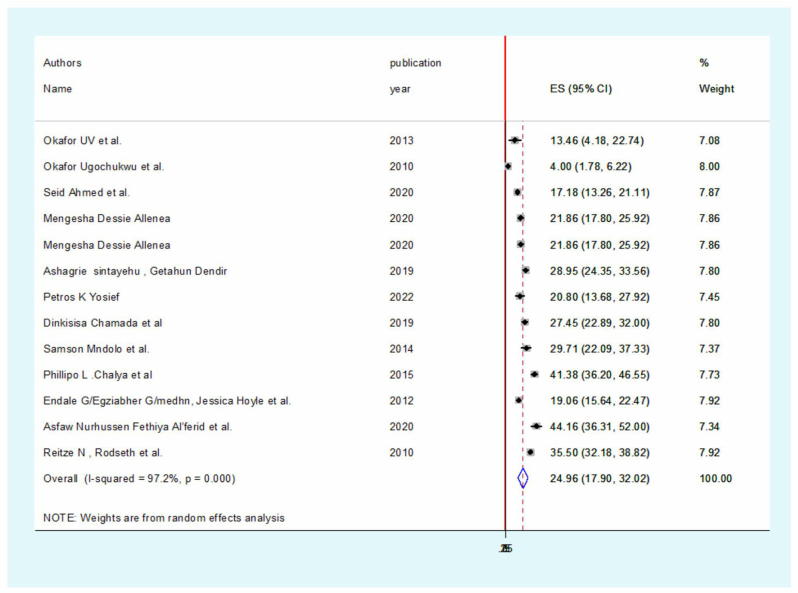
Forest plot showing the pooled incidence of postoperative nausea and vomiting among patients in Africa.

With the random effects model, the continental pooled incidence of postoperative nausea and vomiting among African patients was therefore 24.96% (95% CI: 17.903–32.018), and the heterogeneity index (*I*^2^) was 97.2% (*P* < 0.001) (Fig. 2).

**Figure 3.**
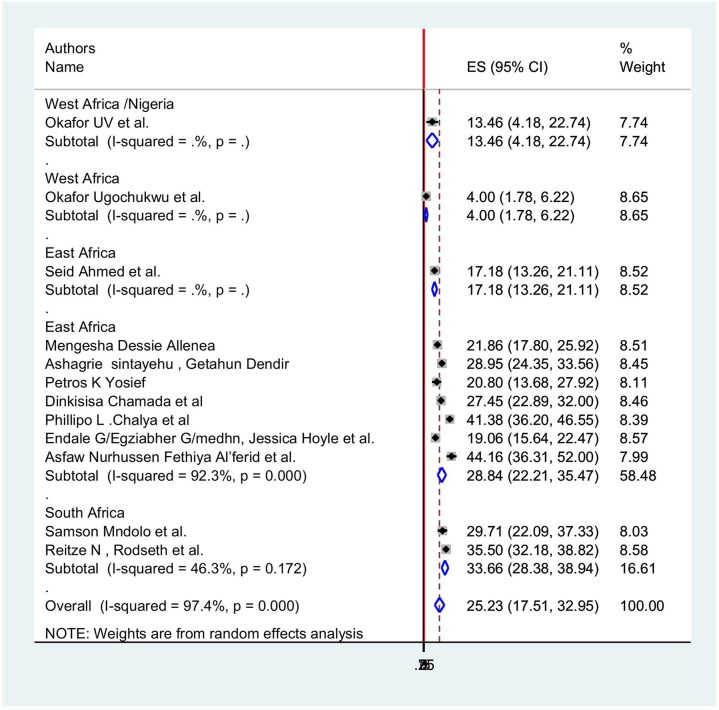
Forest plot showing subgroup analysis postoperative nausea and vomiting in regions of Africa.

According to the results of the subgroup analysis, the regions of South Africa, Ethiopia, and West Africa, Nigeria had the highest and lowest incidences, respectively, of postoperative nausea and vomiting among surgical patients in Africa (33.66%; 95% CI: 28.3, 38.3) and (4%; 95% CI: 1.78, 6.2), *I*^2^ = 97.5% (Fig. 3)

### Heterogeneity and publication bias

**Figure 4.**
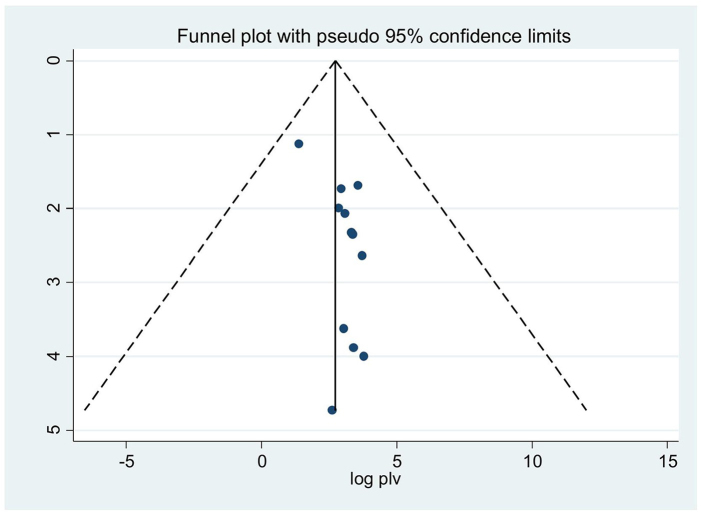
Funnel plot to test the publication bias in 13 studies with 95% confidence limits.

In order to mitigate and reduce the documented variability of this research (*I*^2^ = 98.9%), we employed random effect techniques to forecast effect size. The source of heterogeneity was also found by meta-regression, which took the sample size and the year of publication into account as factors. The outcome shows that the sample size and the year of publication have no bearing on the degree of heterogeneity amongst studies (Table 2). The Egger’s test and a funnel plot are used to visually represent the presence of publication bias. An examination of the funnel plot visually revealed an uneven distribution (Fig. 4), which the Egger test (*P* < 0.05) indicates is statistically significant. Additionally, non-significant (*P* > 0.05) findings from the Beg test demonstrate the existence of publication bias. Additionally, we performed sensitivity analysis by excluding studies step by step to evaluate the effect of a single study on the overall effect estimate. The result indicated that removing a single study did not have a significant influence on pooled prevalence (Table 3). We also performed a subgroup analysis based on the region in Africa.

**Table 2:** Meta-regression analysis of factors affecting between-study heterogeneity.

Heterogeneity source	Coefficients	Std. err.	*P* value
Publication year	0.7176	0.8341	0.410
Sample size	0.0149	0.0184	0.437

**Table 3:** Sensitivity analysis of pooled incidence of postoperative nausea and vomiting for each study being removed one at a time.

Study omitted	Publication year	Estimate [95% CI]
Okafor UV *et al*^[18]^.	2013	15.37 (4.85, 48.68)
Okafor *et al*^[19]^.	2010	24.98 (6.56, 94.98)
Seid Ahmed *et al*^[20]^	2020	15.17 (4.58, 50.19)
Dessie Allenea *et al*^[21]^	2020	14.87 (14.51, 49.025)
Ashagrie Sintayehu, Getahun Dendir^[22]^	2019	14.71 (4.51, 47.93)
Petros K Yosief^[23]^	2020	15.22 (4.77, 48.5)
Dinkisisa Chamada *et al*^[24]^	2019	14.75 (14.52, 48.1)
Samson Mndolo *et al*^[25]^.	2014	15.10 (4.74, 48.05)
Phillipo L .Chalya *et al*	2015	14.57 (4.510, 47.11)
Asfaw Nurhussen Fethiya Al’ferid *et al*^[26]^	2020	14.99 (4.71, 47.65)
Reitze N, Rodseth *et al*	2010	13.69 (4.05, 46.34)
Endale G/Egziabher G/medhn, Jessica Hoyle *et al*^[14]^	2012	14.92 (4.43, 50.26)

### The incidence of postoperative nausea among patients who underwent surgery

To find the pooled effect size, a DerSimonian and Laird random effects model was fitted.

**Figure 5.**
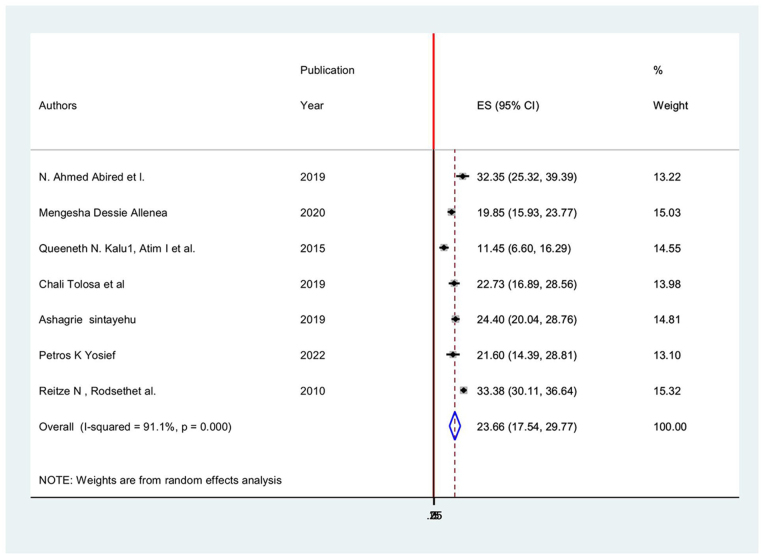
Forest plot showing the pooled incidence of postoperative nausea among patients in Africa.

As a result, using a random effects model, the national pooled incidence of postoperative nausea among patients in Africa was 23.655% (95% CI: 17.542–29.769), and the heterogeneity index (*I*^2^) was 91.1% (*P* < 0.001) (Fig. 5).

**Figure 6.**
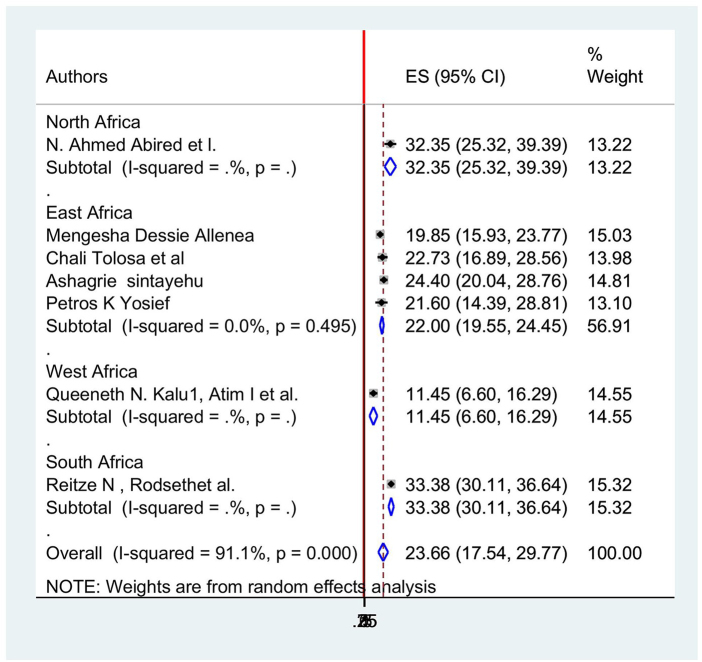
Subgroup analysis of postoperative nausea incidences in different regions of Africa.

The subgroup analysis result showed that the South African, Ethiopian, and West African/Nigerian regions had the highest (33.37%; 95% CI: 30.107, 36.64, *I*^2^ = 91.1%) and lowest (11.44%; 95% CI: 6.603, 1 6.2, *I*^2^ = 91.1%) prevalence of postoperative nausea among surgical patients in Africa, respectively (Table 4 and Fig. 6).

**Table 4:** Subgroup analysis.

Study	ES	[95% Conf.	Interval]	% Weight
North Africa				
N. Ahmed Abired *et al*	32.35	25.32	39.38	13.22
Sub-total				
D + L pooled ES	32.35	25.32	39.38	13.22
East Africa				
Mengesha Dessie Alle	19.849	15.93	23.76	15.03
Chali Tolosa *et al*	22.72	16.89	28.56	13.98
Ashagrie Sintayehu	24.39	20.03	28.75	14.81
Petros K Yosief	21.60	14.38	28.81	13.10
Sub-total				
D + L pooled ES	21.99	19.54	24.45	56.91
West Africa				
Queeneth N. Kalu, A	11.44	6.60	16.28	14.55
Sub-total				
D + L pooled ES	11.44	6.60	16.28	14.55
South Africa				
Reitze N, Rodsethet	33.37	30.10	36.64	15.32
Sub-total				
D + L pooled ES	33.37	30.10	36.64	15.32
Overall				
D + L pooled ES	23.65	17.54	29.76	100.00

### Heterogeneity and publication bias

**Figure 7.**
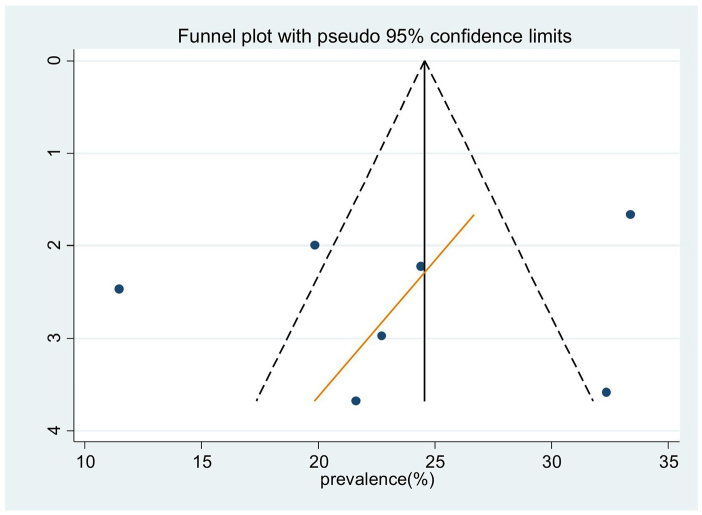
Funnel plot to test the publication bias in seven studies with 95% confidence limits.

In order to mitigate and reduce the observed heterogeneity of this research (*I*^2^ = 98.9%), we conducted a subgroup analysis according to the African region. In order to determine the source of heterogeneity, sample size and the year of publication were also included as factors in a meta-regression analysis. According to Table 5, there is no correlation between the year of publication and sample size and study heterogeneity. The Egger’s test and a funnel plot are used to visually represent the presence of publication bias. A visual examination of the funnel plot revealed an asymmetrical distribution (Fig. 7), which is further supported by the Egger test’s (*P* > 0.05) finding that the distribution is not statistically significant.

**Table 5.** Meta-regression analysis of factors affecting between-study heterogeneity.

Heterogeneity source	Coefficients	Std. err.	*P* value
Publication year	0.4610584	1.15283	0.71
Sample size	0.0227706	0.0193162	0.304

Additionally, the results of the Beg test are not significant (*P* > 0.05), indicating that there is no publishing bias. To ascertain the ultimate effect size, trim and fill analysis utilizing the random effects model was carried out. Nevertheless, the model produced an effect size (24.9) that was comparable. In addition, we performed sensitivity analysis to assess the impact of a single study on the total effect estimate by gradually eliminating studies. The outcome showed that pooled prevalence was not significantly affected by the removal of a single research (Table 6).

**Table 6:** Sensitivity analysis of pooled incidence for each study being removed one at a time.

Study omitted	Publication year	Estimate [95% CI]
N. Ahmed Abired *et al*	2019	22.77 (3.69,140.27)
Mengesha Dessie Allenea	2020	24.24 (3.38,173.86)
Queeneth N. Kalu, Atim I *et al*	2015	25.941 (3.92,171.56)
Chali Tolosa *et al*	2019	23.34 (3.68, 147.83)
Ashagrie Sintayehu, Getahun Dendir	2019	23.07 (3.36, 157.983)
Petros K Yosief	2022	23.39 (3.81, 143.64)
Reitze N, Rodseth *et al*	2010	20.09 (2.48, 162.31)

### The incidence of postoperative vomiting among patients who underwent surgery

To find the pooled effect size, a DerSimonian and Laird random effects model was fitted.

**Figure 8.**
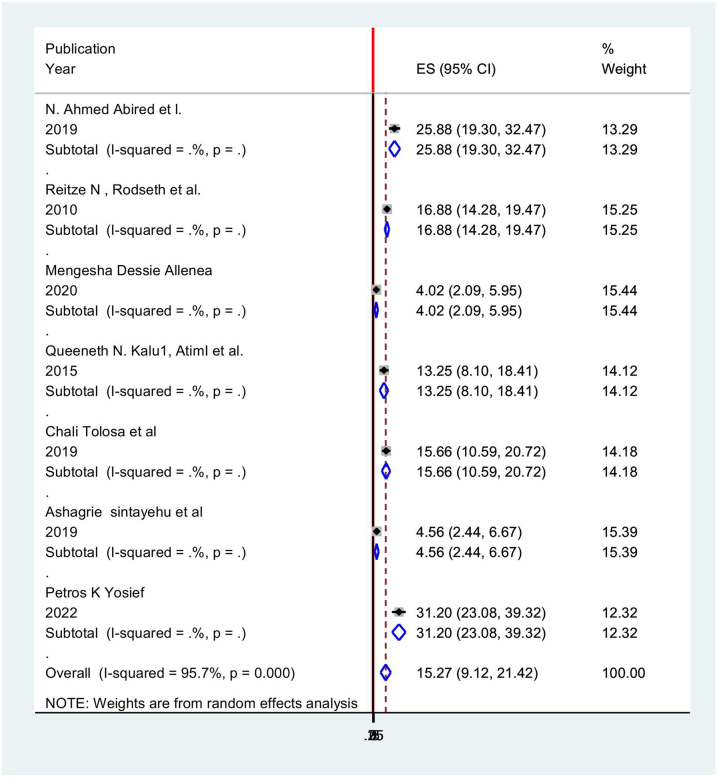
Forest plot showing the pooled prevalence of postoperative vomiting among patients in Africa.

In light of this, the random effects model indicated that the continental pooled incidence of postoperative vomiting among patients in Africa was 15.27% (95% CI: 9.118, 21.424) with a heterogeneity index (*I*^2^) of 95.7% (*P* < 0.001) (Fig. 8).

**Figure 9.**
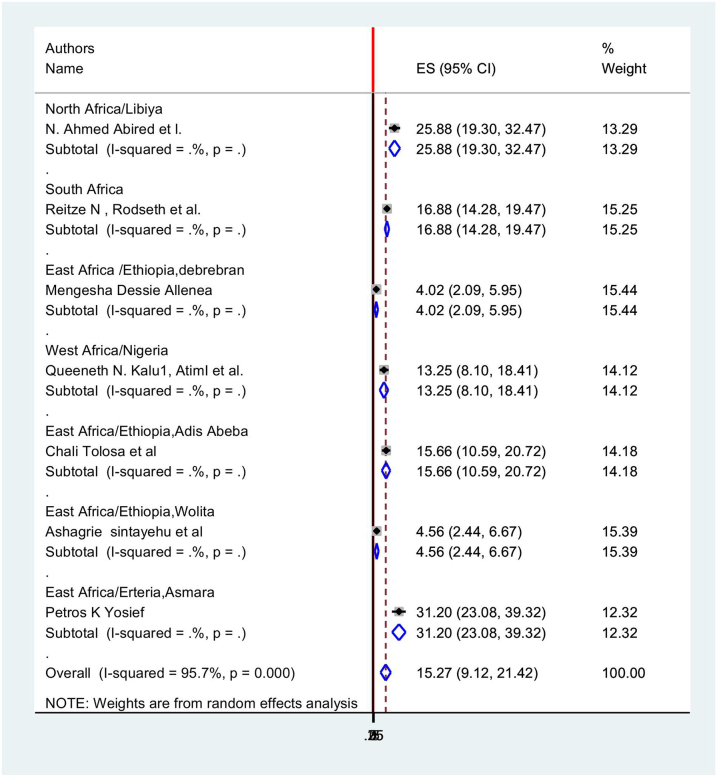
Subgroup analysis of postoperative vomiting among surgery undergone patients in Africa.

According to the results of the subgroup analysis, the regions of East Africa, Ethiopia, and West Africa, Nigeria, respectively, had the highest (31.2%; 95% CI: 23.07, 39.32), and lowest (4.02%; 95% CI: 2.090, 5.950, *I*^2^ = 95.7 %) prevalence of postoperative vomiting among surgical patients in Africa (Table 7 and Fig. 9).

**Table 7:** Subgroup analysis postoperative vomiting among patient in Africa.

Study	ES	[95% Conf.	Interval]	% Weight
North Africa				
N. Ahmed Abired *et al*	25.88	19.29	32.46	13.29
Sub-total				
D + L pooled ES	25.88	19.29	32.46	13.29
East Africa				
Mengesha Dessie Alle	4.02	2.09	5.95	15.44
Chali Tolosa *et al*	15.65	10.59	20.71	14.18
Ashagrie Sintayehu	4.55	2.44	6.67	15.39
Petros K Yosief	31.20	23.07	39.32	12.32
Sub-total				
D + L pooled ES	12.53	5.64	19.43	57.33
West Africa				
Queeneth N. Kalu, A	13.25	8.09	18.41	14.12
Sub-total				
D + L pooled ES	13.25	8.09	18.41	14.12
South Africa				
Reitze N, Rodseth E	16.87	14.28	19.47	15.25
Sub-total				
D + L pooled ES	16.87	14.28	19.47	15.25
Overall				
D + L pooled ES	15.27	9.11	21.42	100.00

### Heterogeneity and publication bias

In order to mitigate and reduce the stated heterogeneity of this study (*I*^2^ = 95.7%), we carried out a subgroup analysis according to African region. In order to determine the source of heterogeneity, sample size and the year of publication were also included as factors in a meta-regression analysis. According to Table 8, there is no correlation between the year of publication and sample size and study heterogeneity.

**Table 8:** Meta-regression analysis of factors affecting between-study heterogeneity.

Heterogeneity source	Coefficients	Std. err.	*P* value
Publication year	−0.2444895	0.2703238	0.417
Sample size	−0.0029289	0.0053156	0.611

**Figure 10.**
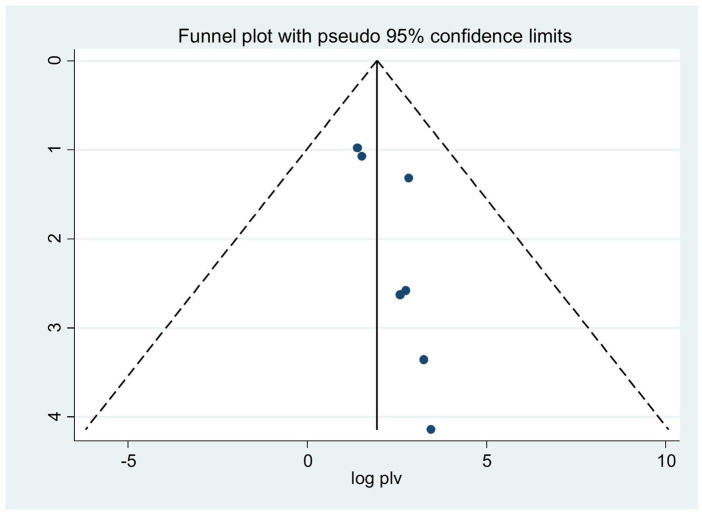
Funnel plot to test the publication bias in seven studies with 95% confidence limits.

The Egger’s test and a funnel plot are used to visually represent the presence of publication bias. A visual examination of the funnel plot revealed an asymmetrical distribution (Fig. 10), which is further supported by the Egger test’s (*P* > 0.05) finding that the distribution is not statistically significant. Additionally, the results of the Beg test are not significant (*P* > 0.05), indicating that there is no publishing bias. To ascertain the ultimate effect size, trim and fill analysis utilizing the random effects model was carried out. Using the model, a different effect size (7.12) was found. In addition, we performed sensitivity analysis to assess the impact of a single study on the total effect estimate by gradually eliminating studies. The outcome showed that the removal of one study had no discernible impact on the pooled prevalence (Table 9).

**Table 9:** Sensitivity analysis of pooled prevalence of postoperative vomiting for each study being removed one at a time.

Study omitted	Publication year	Estimate [95% CI]
N. Ahmed Abired *et al*	2019	6.66 (2.07, 21.44)
Mengesha Dessie Allenea	2020	9.39 (2.23,39.39)
Queeneth N. Kalu, AtimI *et al*	2015	6.714 (2.0621,21.866)
Chali Tolosa *et al*	2019	6.64 (2.03, 21.66)
Ashagrie Sintayehu, Getahun Dendir	2019	8.28 (2.10,32.66)
Petros K Yosief	2022	6.73 (2.106, 21.54)
Reitze N, Rodseth *et al*	2010	5.58 (1.54, 20.18)

### Factors associated with postoperative nausea and vomiting

In order to determine the factors associated with postoperative nausea and vomiting, twelve variables were retrieved. At *P* values of less than 0.05, five of these variables – the kind of operation, the induction anesthetic drug, the prophylaxis of nausea and vomiting, the history of motion sickness, and the history of PONV – were found to be strongly associated with postoperative nausea and vomiting.

Compared to their counterparts, patients with a history of motion sickness and postoperative nausea and vomiting were approximately 3.19 and 4.33 times more likely to experience these side effects [OR: 3.11 (95% CI (1.08–9.42), *P* < 0.036, *I*^2^: 94.1%) and OR: 4.33 (95% CI (2.65–7.07), *P* < 0.001, *I*^2^: 64.9%)].

Additionally, compared to their counterparts, patients who had taken prophylactic medication for nausea and vomiting and those who had a Propofol anesthetic induced were approximately 81% and 42% less likely to experience postoperative nausea and vomiting [OR: 0.194 (95% CI (0.04–0.93)) and OR: 0.585 (95% CI (0.42–0.8))], respectively. Additionally, compared to patients who underwent orthopedic, maxillofacial, urological, gynecologic, or otolaryngology procedures, patients who underwent general surgery, such as abdominal, thyroid, or breast procedures, had a 50% lower risk of developing postoperative nausea and vomiting [OR: 0.5 (95% CI (0.33–0.77))] (Table 10).

**Table 10:** Factors associated with postoperative nausea and vomiting in Africa, 2022.

Determinants	Comparison	Number of studies	Sample size	OR (95% CI)	*P* value	*I*^2^ (%)	Heterogeneity test (*P* value)	Heterogeneity test (*P* value)	Egger test (*P* value)	
Sex^[21,23,24,27,28]^	Female vs male	5	1527	1.343 (0.205–8.800)	0.758	97.8	0.000	0.187	0.171	
Age^[24,28]^	**≤**50 years vs >50 years	2	716	0.636 (0.36–1.12)	0.117	0	0.472	0.351	0.176	
Smoking history^[14,21,23,24,27,28]^	Yes vs no	5	1738	0.721 (0.183–2.833)	0.639	91	0.000	0.308	.
Type of surgery ^[21,24,27-29]^	General surgery vs others	5	1573	1.379 (1.07–1.76)	0.012	89.6	0.000	0.222	0.954	
Anesthesia type^[21,24,28,29]^	GA vs RA	4	1284	0.991 (0.32–3.12)	0.987	92	0.000	0.085	. 0.097	
Duration of surgery^[14,20,21,24,28]^	**≤**1 hour vs >1 hour	5	1876	0.60 (0.28–1.32)	0.205	90	0.000	0.365	0.002	
Induction agent^[21,23,24]^	Propofol vs other IV agent	3	756	0.585 (0.42–0.8)	0.001	0	0.753	0.002	0.092	
Inhalational anesthesia^[22,24]^	Yes vs no	2	746	1.643 (0.784–3.446)	0.189	67.8	0.078	0.208	0.200	
Systemic Opioid^[21,22,24,27,28]^	Yes vs no	5	1746	2.19 (0.47–10.21)	0.39	97	0.000	0.003	0.578	
Prophylaxis^[21,23,24]^	Yes vs no	3	891	0.194 (0.04–0.935)	0.041	92.7	0.000	0.145	0.284	
History of motion sickness^[6,14,20,21,23,24,28]^	Yes vs no	6	1901	3.19 (1.08–9.42)	0.036	94.1	0.000	0.590	0.954	
History of PONV^[14,20-22,24,28]^	Yes vs no	7	2437	4.33 (2.654–7.07)	0.000	64.9	0.009	0.084	0.410	

## Discussion

To study postoperative nausea and vomiting among surgical patients in Africa, a systematic review and meta-analysis were carried out. Postoperative nausea and vomiting were estimated to have been 24.96% (95% CI: 17.903–32.018), 23.655 % (95% CI: 17.542–29.769), and 15.27 % (95% CI: 9.118–21.424) in the pooled estimate. The following are factors related to postoperative study.

Factors such as gender, age, smoking history, surgical procedure, induction agent, inhalation anesthesia, prophylaxis, systemic opioid, motion sickness history, and PONV history.

Our findings are in line with comparable systematic reviews and meta-analyses conducted in Iran, where the prevalence of nausea and vomiting was found to be 27.7%, 31.4%, and 16.8%, respectively. The decrease in incidence could result from genetic population differences caused by geographic differences; sample size understudied could possibly contribute to the difference^[30]^.

One independent risk factor that may influence postoperative nausea and vomiting is the type of operation. Patients who have undergone risky surgeries such as gynecologic, obstetric, ENT, or endocrine have a 1.4 times higher risk of experiencing nausea and vomiting after surgery than patients who have had orthopedic, urological, maxillofacial, or endocrine surgeries. This outcome is comparable to European systematic reviews and meta-analyses^[8]^.

Nevertheless, this data contradicts a meta-analysis finding that shown a higher prevalence of PONV following surgery for breast and abdominal malignancies than following other types of procedures^[30]^. In our analysis, abdominal surgery was categorized with other operations that have a higher risk of causing postoperative nausea and vomiting, which could explain the difference in outcomes.

Propofol induction reduces the risk of postoperative nausea and vomiting by 42%. This result is consistent with a report from another study that compared the effectiveness of inhalational anesthetics and Propofol as intravenous drugs. Propofol may reduce the incidence of postoperative nausea and vomiting because of its antiemetic action on sub-hypnotic dosage induction^[3,31,32]^.

Compared to their counterparts, patients with a history of motion sickness had a 3.2 times higher likelihood of experiencing postoperative nausea and vomiting. This finding is consistent with results from a main study that was published after our data gathering period.

Patients who receive prophylactic medication for nausea and vomiting have a 42% decreased risk of experiencing nausea and vomiting during the postoperative phase, which is consistent with a German study’s findings. This could be because patients who receive prophylactic medication for nausea and vomiting have a lower incidence of nausea and vomiting during the postoperative phase ^[33]^.

Strengths of this study: It is the first systematic review and meta-analysis to the best of our knowledge on the prevalence and risk factors for postoperative nausea and vomiting in Africa. A high-standard JBI MAStARI (Meta-Analysis Statistical Assessment and Review Instrument) was employed by the assessors. This study’s limitation is that several other research lacked enough predictor factors to accurately assess the degree of prediction The lack of continental representativeness stems from the fact that no data from the northern regions of Africa was found, despite the fact that the demographic characteristics of the population in the North African region were nearly identical to those of the other regions. Nevertheless, efforts were made to include all other potential variables that occurred across the identified database.

## Conclusion

This review revealed that research participants have greater rates of postoperative nausea and vomiting. The following were the independent predictors of postoperative nausea: Surgery type, anesthetic medication type, antiemetic prophylaxis, motion sickness history, prior postoperative nausea and vomiting history.

With a focus on surgery lists such as abdominal, gynecologic, ENT, maxillofacial, history of motion sickness, and history of postoperative nausea patient, there should also be an urgent clinical measure to prevent, detect, and offer prophylactic to avoid postoperative nausea. If the patient’s clinical state permits, total intravenous anesthesia based on Propofol may be used during surgery.

## Data Availability

If necessary request to the corresponding author to access and use the data, it can be obtained

## Registration of systematic reviews

This review was registered in the Prospero database with reference number CRD 344024.
